# Prise en charge des plaies du périnée en post partum: faut-il prescrire systématiquement un antibiotique?

**DOI:** 10.11604/pamj.2017.28.144.12915

**Published:** 2017-10-16

**Authors:** Florent Ymele Fouelifack, Filbert Eko Eko, Claude Odile Vanessa Ebode Ko’A, Jeanne Hortence Fouedjio, Robinson Enow Mbu

**Affiliations:** 1 Département de Gynécologie et Obstétriques de l’Institut Supérieur de Technologie Médicale de Nkolondom à Yaoundé, Unité de Gynécologie et Obstétriques de l’Hôpital Central de Yaoundé, Cameroun; 2 Institut Supérieur de Technologie Médicale de Nkolondom à Yaoundé, Faculté de Médecine et des Sciences Biomédicales de l’Université de Yaoundé I, Cameroun; 3 Département de Gynécologie et Obstétriques de la Faculté de Médecine et des Sciences Biomédicales de l’Université de Yaoundé I, Unité de Gynécologie et d’Obstétrique de l’Hôpital Central de Yaoundé, Cameroun

**Keywords:** Efficacité, antibiotique, déchirures, épisiotomie, plaies périnéales, prise en charge, Effectiveness, antibiotic, tears, episiotomy, perineal wounds, treatment

## Abstract

**Introduction:**

L'intégrité des voies génitales n'est pas toujours respectée au cours de l'accouchement. Aucun protocole de prise en charge des plaies périnéales du post-partum (déchirures et épisiotomies) n'existant dans nos services, la prise en charge reste praticien dépendant. Nous avons entrepris cette étude dans le but d'établir l'intérêt de l'antibiotique dans le traitement des plaies périnéales du post-partum. Notre objectif était d'évaluer l'impact de l'antibioprophylaxie sur le processus de cicatrisation et dans la prévention des complications infectieuses après réparation.

**Méthodes:**

Il s'agissait d'une étude de cohorte prospective sur une durée de 6 mois, soit du 1er janvier au 31 mai 2016, menée dans l'Unité de Gynécologie et Obstétriques de l'Hôpital Central de Yaoundé. Deux groupes A et B d'accouchées avec déchirure périnéale et/ou épisiotomie étaient suivies. Le groupe A était composé de 85 accouchées à qui on avait prescrit le protocole compresse imbibée de Bétadine^®^ (placebo). Le groupe B (ou groupe test) était composé d'accouchées qui en plus du placebo, avaient la prescription d'un antibiotique (association 1000mg d'amoxicilline et 125mg d'acide clavulanique à raison de 1000mg 2fois par jour par voie orale pendant 05 jours). Les 2 groupes étaient suivis à J0, J2 et J9. Nos critères d'évaluation de la prise en charge étaient: la douleur, l'infection, la tuméfaction, la propreté de la plaies et le delai de cicatrisation complete. Les données étaient saisies et analysées à l'aide des logiciels Epidata analysis version 3.2 et STATA version 12.0 (Texas USA 2001). Les corrélations entre les variables étaient recherchées selon le cas par le chi carré, l'Odds ratio et avec la valeur de P (significatif pour toute valeur ≤ 0.05).

**Résultats:**

La moyenne d'âge était de 26.32 ± 6.5 ans avec des extrêmes de 15 et 43 ans. Les primipares représentaient 55.9% de la population d'étude. La douleur représentait le principal symptôme à J0 post-partum sans prédominance significative d'un groupe (OR = 0.9; IC = 0.14-7.19; P = 1). Les plaies tuméfiées étaient la deuxième plainte sans variation significative dans les deux groupes (OR = 1.69 ; IC = 0.88-3.24 ; P = 0.13). Aucune variation significative n'a été observée à J0, J2 et J9 entre les 2 protocoles en ce qui concerne les indicateurs étudiés : évolution de la douleur, l'infection, la tuméfaction et le délai de cicatrisation. A J9 la guérison était complète dans les deux groupes et les 2 protocoles s'équivalaient au niveau de l'efficacité et la prévention des infections.

**Conclusion:**

Au terme de cette étude les deux protocoles étaient équivalents. Nous concluons que pour l'intérêt de l'économie de la santé, il n'est pas utile de prescrire les antibiotiques pour la prise en charge des plaies périnéales.

## Abstract (en)

**Introduction:**

Genital tract integrity is not always mantained during childbirth. No treatment protocol for post-partum perineal wounds (tears and episiotomies) existed in our Department, thus their management depends upon clinician. This study aimed to establish the role of antibiotic in the treatment of post-partum perineal wounds as well as the impact of antibioprophylaxis on wound healing and on prevention of infectious complications after repair.

**Methods:**

We conducted a prospective cohort study in the Department of Gynecology and Obstetrics at the Central Hospital of Yaoundé over a period of 6 months, from 1 January to 31 May 2016. Post-partum women with perineal tear and/or episiotomy were divided into two groups (A and B) and followed up. The group A was composed by 85 post-partum women under treatment protocol based on compresses soaked in Betadine^®^ (placebo). The Group B (or test group) was composed of post-partum women who had received placebo plus antibiotic (oral amoxicillin/clavulanic acid 875 mg/125 mg twice a day for 05 days). Both groups were followed up on day 0, day 2 and day 9. Our criteria for the evaluation of treatment were: pain, infection, swelling, cleanliness of the wounds and average healing time. Data were collected and analyzed using the software Epidata analysis version 3.2 and STATA version 12.0 (Texas USA 2001). The correlations between the variables were identified by chi-square, odds ratio and p value (using any p-value ≤ 0.05 as statistical significance cut-off) according to the case.

**Results:**

The average age was 26.32 ± 6.5 years, ranging between 15 and 43 years. Primiparous women accouted for 55.9% of the study population. At day 0 post-partum the main symptom was pain, without significant predominance of a group (OR = 0.9; CI = 0.14-7.19; p = 1). Swollen wounds were the second complaint, without significant variation between the two groups (OR = 1.69; CI = 0.88-3.24; p = 0.13). At day 0, day 2 and day 9 no significant variation was observed between the two treatment protocols with regard to the indicators analyzed: pain evolution, infection, swelling and average healing time. At day 9 the healing was complete in both groups and the two treatment protocols were shown to be equivalent in their effectiveness and prevention of infections.

**Conclusion:**

At the end of this study the two treatment protocols were shown to be equivalent. Antibiotics should not be prescribed for perineal wounds in order to contain the healthcare costs.

## Introduction

Les déchirures obstétricales sont des lésions résultant d'une solution de continuité survenant au décours de l'accouchement et intéressant le périnée, la vulve, le vagin et/ou le col de l'utérus [1]. Les déchirures périnéales demeurent quelle que soit la région du monde ou l´hôpital considéré, l'un des traumatismes obstétricaux les plus fréquents [2, 3]. Elles sont observées dans 20 à 60% des accouchements, dont 75% lors du premier accouchement [4-6]. Au Cameroun, le taux varie de 8 à 13.5% [7-10]. Qu'il s'agisse de déchirures ou d'épisiotomie, la réparation minutieuse est impérative [11-14]. L'infection des lésions périnéales de l'accouchement est une menace quasi-constante en raison de la septicité naturelle du vagin et de la présence des lochies qui constituent un excellent milieu de culture pour les germes aussi saprophytes qu'exogènes [15,16], d'où l'importance de la mise en place de mesures d'asepsie périnéale dans les suites de couche [15]. Le bain de siège, méthode introduite par Louis Kuhne [17], à la fin du 19^ème^ siècle, est l'un des moyens efficaces pour soulager la douleur, diminuer le risque infectieux et l'inconfort associé à une affection douloureuse dans la région pelvienne. Ainsi, si cette mesure d'asepsie n'est pas faite en post-partum, cela peut avoir un impact sur l'activité quotidienne d'une femme et même sur sa relation avec son bébé et son partenaire [18]. C'est pourquoi la prise en charge post-réparation devrait être codifiée en tenant compte l'intérêt et du bénéfice du traitement. Dans notre formation hospitalière, il n'existe aucun consensus concernant la prise en charge des plaies périnéales en post-partum. Dès lors, la prise en charge est praticien dépendante. En fonction du praticien on observe 3 protocoles de prise en charge dans le service: le premier, le plus ancien est le bain de siège au polyvidone iodé (Bétadine bleue^®^), qui est progressivement abandonné dans le service au profit du deuxième protocole fait de compresse imbibé de polyvidone iodé depuis qu'une étude faite par Eko et coll en 2009 dans le même service [8] avait montré une meilleure compliance au traitement par rapport au bain de siège, avec des résultats équivalents. Le 3^e^ protocole prescrit dans le service est celui qui associe à l'un des 2 précédemment cités un antibiotique par voie orale. Dans la littérature, nous n'avons pas trouvé d'article mettant pas clairement en évidence le bénéfice de l'antibiotique dans la prise en charge des déchirures périnéales et/ou épisiotomies. Cette étude est réalisée dans le but d'établir l'intérêt de l'antibiotique dans le traitement des plaies périnéales du post-partum. Notre objectif général était d'évaluer le bénéfice de l'antibiotique sur la prise en charge des plaies périnéales après suture réparatrice en post partum. Plus spécifiquement de comparer le taux d'infection, le délai de cicatrisation et la survenue de complications selon que le protocole utilisé contient un antibiotique ou non.

## Méthodes

Nous avons mené une étude de cohorte prospective, dans l'Unité de Gynécologie et Obstétriques de l'Hôpital Central de Yaoundé (HCY), sur une durée de 6 mois, soit du 1^er^ janvier 2016 à 31 juin 2016. Cet unité à une capacité totale de 65 lits, constitue un centre de référence national et contient en son sein la plus grande maternité de la région du centre, qui réalise plus de 300 accouchements par mois. Notre population d'étude était constituée de toutes les femmes ayant accouché par voie basse pendant la période d'étude. Etaient inclues, toutes les accouchées ayant eu une déchirure du périnée et/ou une épisiotomie, qu'elles soient ou non sous antibiotique (prescrite dans un but de prise en charge de sa plaie périnéale). Etaient exclues toutes les femmes présentant des facteurs de risques infectieux tels: rupture prématurée et/ou prolongée des membranes, accouchement septique, infections urinaires, pneumopathie, infection systémique, diabète, immunodépression (VIH, diabète, corticothérapie au long court, lupus) et celles n'ayant pas donné leur consentement, les femmes sous antibiotique avant l'accouchement, les femmes perdues de vue ou n'ayant pas respecté le protocole de traitement. La taille minimale de chaque groupe était calculée par la formule de Schlesselman suivante: T= (1/1-f) x [2(Za+Zß)^2^ x p(1-p)] / (p0-p1)^2^.

Où T = taille de l'échantillon de chaque groupe, f = nombre de cas perdu de vue, P = proportion de sujets exposés dans les 2 groupes cas et témoins, α = risque de type I, β = risque de type II, P_0_ = Proportion de t émoin exposé, P1 = Proportion de cas exposé et p = (p1+p0) / 2.

Pour un intervalle de confiance à 95% on aura un risque α = 5%; Zα = constante = 1.96. Nous obtenons ainsi une taille minimale de 79 femmes par groupe, mais pour augmenter la validité de notre étude, nous avons recruté 85 par groupe. Le counseling aux femmes commençait dès leur entrée en salle de travail, continuait pendant le travail et en postpartum immédiat. Apres consentement nous les recrutions en postpartum immédiat si elles avaient eu une déchirure périnéale et/ou une épisiotomie au cours de l'accouchement et si nos critères de sélection étaient respectés. Dès lors la femme était recrutée et on commençait à remplir la fiche technique. Un interrogatoire sommaire était fait pour rechercher l'anamnèse (caractéristiques sociodémographique et obstétricales, le risque infectieux) de l'accouchée. On attendait ensuite que le consultant après suture du périnée fasse sa prescription, ce qui nous permettait de classer la femme dans un des groupes A ou B. Il faut noter ici que les participantes n'étaient recrutées qu'après la prescription de leur traitement par un consultant. Aucun consultant traitant n'intervenait dans l'etude. Nous ne recrutions que celles qui étaient sous l'un des protocoles sans toutefois intervenir dans la prescription. Nous n'avons donc fait aucune randomisation et n'avions eu aucune influence sur la prescription des praticiens.

Le protocole A consistait à faire une toilette périnéale à l'eau propre avant de prendre une compresse stérile imbibée de polyvidone iodé (Bétadine Bleu^®^) et la poser sur le site de la plaie trois fois par jour et après chaque selle. Le protocole B consistait à ajouter au protocole A un antibiotique (association amoxicilline et acide clavulanique 1 gramme deux fois par jour pendant 5 jours) par voie orale. Les accouchées étaient suivies dans le temps, le jour de l'accouchement (J0), le 2^e^ jour (J2) du post partum. Puis un rendez-vous était pris à la sortie (J2) pour le 9^e^ jour (J9) du post-partum pour contrôle et réévaluation des différents indicateurs. Les variables étudiées étaient le type de déchirure périnéale, le protocole reçu, la douleur, l'infection, tuméfaction, la cicatrisation des plaies avec le temps, et les complications. La douleur était évaluée par échelle visuelle analogique (EVA), cotée de 0 à 10 (nous avions expliqué aux femmes que 0 signifiait absence de douleur et 10 signifiait douleur insupportable). Elles devaient nous donner le chiffre qui correspondait à sa douleur durant les jours de suivi. L'inflammation était évaluée par la présence ou l'absence de rougeur et de tuméfaction, l'infection par la présence ou l'absence de suppuration. L'efficacité de l'antibiotique était évaluée sur le délai de cicatrisation, le délai de régression de la douleur et de l'inflammation, enfin par l'absence ou la présence d'infection. Les données compilées sur des fiches techniques étaient saisies grâce au logiciel epidata entry version 3.1 puis analysées grâce aux logiciels epidata analysis version 3.2 et STATA version 12.0 (Texas USA 2001). Les outils statistiques utiles pour l'analyse des résultats étaient: la moyenne, la fréquence, l'écart type, le test de khi-carré et d'autres tests statistiques classiques. Les corrélations entre les variables étaient recherchées selon le cas par le chi carré de Pearson corrigé par le test exact de Fischer, le rapport de cotes (Odds ratio) et la probabilité P. P était considérée significative pour toute valeur inférieure à 0.05. L'association entre les variables était recherchée à l'aide du rapport de cotes exprimé avec un intervalle de confiance à 95%. Une clairance éthique était obtenue auprès du comité d'éthique de la Faculté de Médecine de Science Pharmaceutique de Douala. Une autorisation de mener l'étude était obtenue auprès de l'administration de l'HCY. Les informations recueillies auprès des parturientes étaient traitées avec la plus grande confidentialité et ne servaient qu'à l'étude.

## Résultats

**Caractéristiques générales de la population**: Au cours de la période d'étude, 1599 accouchements par voie basse ont été réalisés. L'âge moyen des accouchées était de 26.32 ± 6.5 ans avec des extrêmes de 15 et 43 ans, la parité moyenne de 1.77. Un total de 187 déchirures et/ou épisiotomies était observé, soit une fréquence des plaies périnéales de 11.69% au décours des accouchements. Parmi ces 187 accouchées avec plaie périnéale, nous avons suivi 170 (2 groupes de 85), dont 26 épisiotomies (soit 8.2% de la population) et 147 déchirures (91.8%). Des 147 déchirures périnéales, 135 (soit 91.8%) étaient du 1er degré, 11(soit 7.5%) du 2e degré et 1seul cas (soit 1.4%) de 3^e^ degré. La comparaison de l'efficacité des 2 protocoles A et B est illustrée par les tableaux et figures ci-dessous.

### Paramètres socio-démographiques

**Répartition des deux groupes A et B par tranches d'âge, le statut matrimonial, le niveau d'étude et la profession**: La répartition des deux groupes A et B par tranches d'âge, statut matrimonial, niveau d'étude et la profession est représentée dans le Tableau 1. Les deux groupes étaient comparables au début de l'étude.

**Tableau 1:** Répartition des deux groupes A et B par tranches d’âge, statut matrimonial, niveau d’étude et de la profession

Caractéristiques	A N = 85(%)	B N = 85 (%)	Total N=170(%)	OR	IC 95%	P value
**Tranches d’âge (ans)**						
≤ 19	9(10,6)	14(16,5)	23(13,5)			
20 – 29	47(55,3)	42(49,4)	89(52,4)	0,67	(0,27-1,66)	0,3830
30 – 39	28(32,9)	28(32,9)	56(32,9)	1,03	(0,53-2,02)	0,9208
≥ 40	1(1,2)	1(1.2)	2(1,2)	1,07	(0,06-17,68)	0,9615
**Statut matrimonial**						
Mariée	28(32,9)	24(28,2)	52(30,6)			
Célibataire	57(48,3)	61(51,7)	118(68,8)	1,25	(0,65-2,40)	0,5055
**Niveau d’étude**						
Primaire	11(12,9)	9(10,6)	20(11,8)			
Secondaire	41(48.2)	39(45,9)	80(47,1)	1,16	(0,43-3,11)	0,7640
Universitaire	33(38,8)	37(43,5)	70(41,2)	1,18	(0,62-2,24)	0,6157
**Profession**						
Fonctionnaire	14(16,5)	18(21,2)	32(18,8)	0,85	(0,37-1,97)	0,7024
Ménagère	23(27,1)	18(21,2)	41(24,1)	1,39	(0,64-3,03)	0,4014
Elève/Etudiante	33(38,8)	36(42,4)	69(40,6)	1,19	(0,46-3,08)	0,7174
Commerçante	10(11,8)	13(15,3)	23(13,5)			
Autres	5(5,9)	0(0,0)	5(2,9)			

**Profil obstétrical**: Le profil obstétrical des 2 groupes est représenté dans le Tableau 2. Le profil obstétrical des deux groupes était comparable au début de notre étude.

**Tableau 2:** Le profil obstétrical des 2 groupes A et B

Caractéristiques	A N = 85 (%)	B N = 85 (%)	Total N=170 (%)	OR	IC 95%	P value
**Gravidité**						
Primigeste	39(45,9)	37(44,0)	76(45,0)	1,08	(0,59-1,97)	0,8105
Multigeste	47(50,5)	46(49,5)	93(55,0)			
**Parité**						
Nullipare	2(2,4)	3(3,5)	5(2,9)	1,08	(0,59-1,97)	0,8105
Primipare	50(58,8)	45(52,9)	95(55,9)	0,60	(0,10-3,76)	0,5816
Paucipare	7(4,1)	39(22,9)	47(27,6)	1,64	(0,81-3,32)	0,1709
Multipare	2(1,2)	20(11,8)	22(12,9)	0,77	(0,30-1,97)	0,5839
Grande multipare	1(0,6)	0(0,0)	1(0,6)	0,00	(0,0-0,0)	0,00

**Efficacité du traitement antibiotique:** Les différentes variables utilisées pour apprécier l'efficacité du protocole avec antibiotique figurent dans le Tableau 3. Concernant l'ensemble des variables, aucun protocole n'était plus efficace.

**Tableau 3:** Efficacité du protocole avec antibiotique

Variables	A N=85 (%)	B N=85 (%)	Total N=170(%)	OR	IC	P value
**Douleur**						
J0	28(32,9)	38(44,7)	66(38,8)			
J2	2(1,2)	3(1,8)	5(2,9)	0,99	(0,14-7,19)	1
J9	0(0,0)	0(0,0)	0(0,0)			
**Inflammation / œdème**						
J0	11(12,9)	10(11,8)	21(12,4)			
J2	0(0,0)	2(2,4)	2(1,2)	1,69	(0,88-3,24)	0,13
J9	0(0,0)	0(0,0)	0(0,0)			
**Infection**						
J0	0(0,0)	0(0,0)	0(0,0)			
J2	0(0,0)	0(0,0)	0(0,0)			
J9	0(0,0)	0(0,0)	0(0,0)			
**Propre**						
J0	46(54,1)	37(43,5)	83(48,8)			
J2	83(97,6)	80(94,1)	163(95,9)	1,3	(0,43-2,95)	0,8
J9	0(0,0)	0(0,0)	0(0,0)			
**Guérisoncomplète**						
J0	0(0,0)	0(0,0)	0(0,0)			
J2	0(0,0)	0(0,0)	0(0,0)0			
J9	85(100,0)	85(100,0)	170(100,0)			
							

### Représentations graphiques de l'évolution de chaque variable en fonction du temps

**Figure 1:**
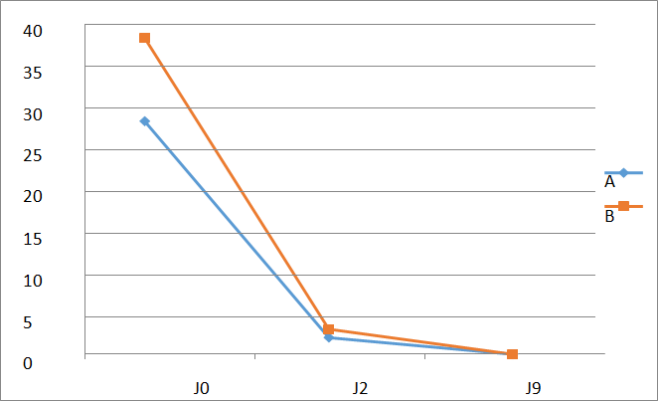
Courbe d’évolution de la douleur dans le temps

**La Douleur:** L'évolution de la douleur dans les deux groupes est représentée par la Figure 1. A J0 post-partum la douleur représentait le maitre symptôme dans les 2 groupes. De J2 à J9 du post-partum on notait une régression significative de la douleur dans les deux groupes. A J9, aucune accouchée ne se plaignait de douleur périnéale dans les deux groupes.

**Figure 2:**
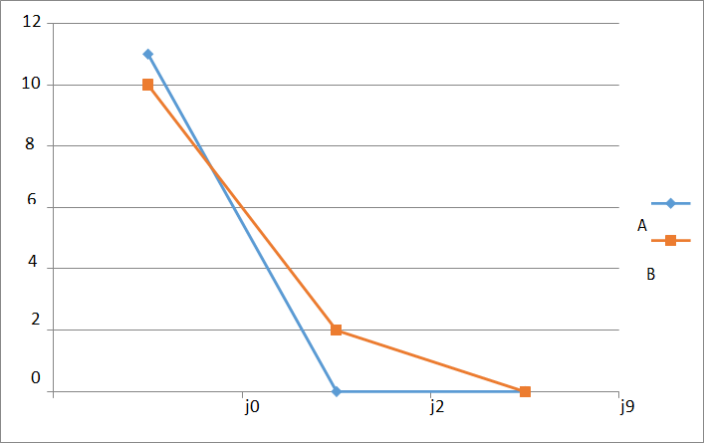
Courbe d’évolution de l’inflammation avec le temps

**L'inflammation**: L'évolution de l'inflammation dans les deux groupes est représentée par la Figure 2. De J0 a J2, on notait une disparition rapide de l'inflammation dans les 2 groupes. A J9 aucune inflammation ou tuméfaction n'avait été retrouvée dans les deux groupes.

**L'infection**: Nous n'avons retrouvé aucun cas d'infection durant le suivi post-partum quel que soit le protocole de traitement utilisé.

**Figure 3:**
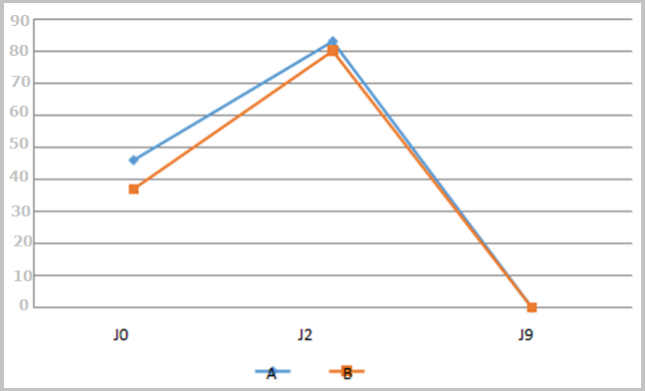
Courbe de propreté de la plaie dans le temps

**Propreté de la plaie**: L'évolution de ce paramètre est representée par la Figure 3. De J0 a J9, on note que les courbes de propreté des plaies sont superposables dans les 2 groupes.

**Figure 4:**
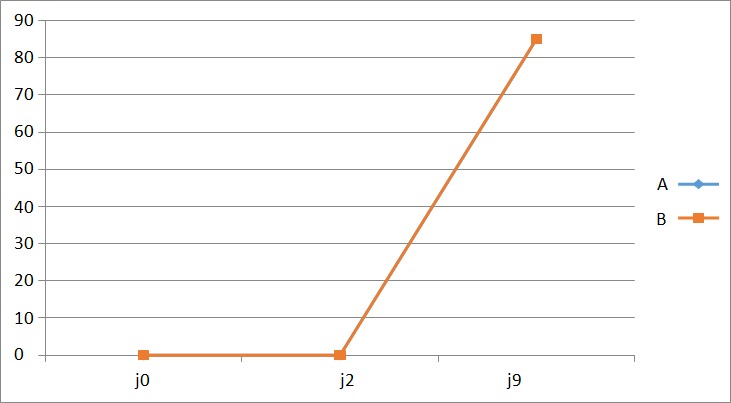
courbe de guérison complète

**Guérison complète**: La guérison complète dans le temps est représentée par la Figure 4. Les 2 courbes étaient superposables. A J9, on notait une guérison complète dans les 2 groupes.

## Discussion

**Facteur limitant**: N'ayant pas bénéficié d'un financement ou d'une dotation en médicaments, nous n'avons pas pu mener une étude expérimentale randomisée qui aurait été le modèle idéal pour rechercher l'efficacité thérapeutique d'un médicament. Nous avons mené une étude d´observation épidémiologique (cohorte) car plus réaliste dans nos conditions d'exercice.

**Caractéristiques générales de la population d'étude**: Notre fréquence de 11.69% de plaies périnéales (187 cas /1599 accouchements par voie basse) est inférieure à celle de de 15.7% retrouvée dans le même service en 2012 [9]. La différence serait due au fait que sa durée d'étude était de 2 ans et la taille de son échantillon était plus grande (5997 accouchements par voie basse). La moyenne d'âge était de 26.36 ± 6.4 ans avec des extrêmes de 15 à 43 ans, résultat proche de ceux retrouvés dans plusieurs études [7, 10, 15, 19].

**Profil socio-démographique, profil obstétrical des participantes:** Au début des analyses, les deux groupes étaient comparables. Cette absence de différence significative entre les deux groupes A et B, en ce qui concernait le profil sociodémographique (Tableau 1), et le profil obstétrical (Tableau 2) augmente la fiabilité de nos résultats sur l'efficacité des 2 protocoles.

**Efficacité de l'antibiotique:** Le Tableau 3 nous permet de constater que les 2 protocoles se valent au terme de la prise en charge des plaies périnéales. Quant à la représentation graphique des variables étudiées, nous constatons que dans l'ensemble, les courbes montrant l'évolution de la douleur (Figure 1), de l'inflammation (Figure 2), de la propreté des plaies (Figure 3) et du délai de guérison complète (Figure 4) dans les deux groupes étaient superposables. Dans l'étude de Eko et al, la guérison complète des deux groupes était établie à J9 [8]. Dans la littérature, très peu d'auteurs ont étudié l'effet des antibiotiques dans la prise en charge des plaies périnéales en post partum. Quelques études ont montré un bénéfice des antibiotiques dans la réduction des complications des déchirures périnéales du troisième et du quatrième degré lors de l´accouchement par voie vaginale [18, 20]. Leurs études ne concernaient que les déchirures de 3e et 4e degré, contrairement à la nôtre qui était dominée par les épisiotomies et déchirures de 1er et 2e degré. Cependant, comme dans notre étude, Liabsuetrakul et coll [21] n'ont trouvé aucun avantage à utiliser les antibiotiques en général dans les suites d'accouchements par voie basse. Nous n'avons trouvé aucun avantage à utiliser les antibiotiques dans notre étude.

## Conclusion

L'antibiotique n'a pas montré d'avantage au cours de notre étude dans la prise en charge des plaies périnéales en post partum. Nous pensons que pour l'intérêt de l'économie de la santé, il n'est pas utile de prescrire les antibiotiques pour la prise en charge des plaies périnéales en post partum.

### Etat des connaissances actuelle sur le sujet

- L'effet des antibiotiques dans la prise en charge des plaies périnéales du post partum n'est pas connu.

### Contribution de notre étude à la connaissance

- L'antibiotique n'apporte aucun effet bénéfique dans la prise en charge des plaies périnéales du post partum.

## Conflits d’intérêts

Les auteurs ne déclarent aucun conflit d'intérêts.

## Remerciements

Nous remercions l'administration de l'HCY pour avoir donné l'autorisation de réaliser cette étude, ainsi que le personnel de l'Unité de Gynécologie et Obstétriques et les participantes pour avoir facilité sa réalisation.
